# Effects of Auricular Acupressure on Body Weight Parameters in Patients with Chronic Schizophrenia

**DOI:** 10.1155/2012/151737

**Published:** 2012-09-10

**Authors:** Han-Yi Ching, Shang-Liang Wu, Wen-Chi Chen, Ching-Liang Hsieh

**Affiliations:** ^1^Graduate Institute of Integrated Medicine, College of Chinese Medicine, China Medical University, Taichung 40402, Taiwan; ^2^Department of Psychiatry, Tsao-Tun Psychiatric Center, Department of Health, Executive Yuan, Nan-Tou 54249, Taiwan; ^3^Taiwan Research Association of Health Care, Taichung 40343, Taiwan; ^4^Acupuncture Research Center, China Medical University, Taichung 40402, Taiwan; ^5^Department of Chinese Medicine, China Medical University Hospital, Taichung 40402, Taiwan

## Abstract

Auricular acupressure is widely used in complementary and alternative medicine to reduce body weight, but little is known about the effects of auricular acupressure on body weight parameters in patients with chronic schizophrenia. The purpose of this study was to evaluate the effects of auricular acupressure on body weight parameters in patients with chronic schizophrenia. Eighty-six inpatients with schizophrenia were recruited from chronic wards in a psychiatric center. The participants were randomly divided into experimental (acupressure at 4 acupuncture sites: hunger, stomach, shenmen and endocrine) and control groups, and body weight parameters were determined weekly for 8 weeks. There was no significant difference between the experimental and control groups in mean body weight, waist circumference, or body fat percentage at the pretest or during the entire 8-week study period. Therefore, auricular acupressure did not cause body weight reduction in patients with chronic schizophrenia.

## 1. Introduction

Schizophrenia is a severe mental illness with a chronic course. The diagnosis of schizophrenia, excluding schizoaffective or mood disorder, substance use or general medical condition, and pervasive developmental disorder, is defined by the Diagnostic and Statistical Manual of Mental Disorders, 4th Edition (DSM-IV) as the presentation of positive and negative symptoms for ≥1 month (or less if successfully treated) and deterioration of interpersonal and occupational relations for ≥6 months [1]. The positive symptoms include delusions, hallucinations, disorganized speech, and disorganized or catatonic behavior, and the negative symptoms include affective flattening, alogia, social withdrawal, and the lack of spontaneity.

Patients with schizophrenia have an increased prevalence of obesity and unfavorable body composition compared with the general population [2–4]. The prevalence of obesity among patients with schizophrenia is increasing each year [5–9].

Obesity is a major risk factor for type 2 diabetes, metabolic syndrome, and cardiovascular disease [10–16]. Obesity also has become a major concern in the treatment of mental disorders because it may adversely affect treatment adherence and relapse rates [17, 18].

Furthermore, obesity is associated with reduced quality of life [19], social stigma [20], and greater morbidity and mortality [21]. The United States National Institute of Mental Health convened a meeting in October 2005 and concluded that obesity among individuals with mental disorders has not received adequate research attention [22].

Auricular acupressure is a simple, self-manipulated treatment method that applies *vaccaria* seeds or steel beads to the ear to stimulate auricular acupoints. It is widely used in complementary and alternative medicine to reduce body weight, but little is known about its effect on weight reduction. Previous animal studies suggest that stimulation of the auricular regions is associated with the ventromedial hypothalamus, which affects the satiety center and leads to weight loss [23]. Needle point stimulation on auricular acupoints may increase the secretion of ghrelin, which is a peptide hormone that induces satiation and is inversely related to caloric intake [24]. The purpose of this controlled, single-blind study with stratified random sampling was to explore the effects of auricular acupressure on body weight parameters, including body weight, waist circumference, and body fat percentage, in patients with chronic schizophrenia.

## 2. Methods

The study protocol was approved by the ethics committee of the Tsao-Tun Psychiatric Center Institutional Review Board (TTPC IRB99002) in February 2010, and carried out in compliance with the Declaration of Helsinki. Volunteers were recruited through posters placed in chronic psychiatric wards, all of them were hospitalized Chinese schizophrenia patients. Protocol contents were thoroughly explained to each patient by the investigator. Patients were told that they could withdraw from the study anytime if they were not willing to continue. After patients and their families agreed and signed the informed consent forms, the patients were given the “precautions for auricular acupressure for weight reduction” and related health education pamphlets.

**Figure 1:**
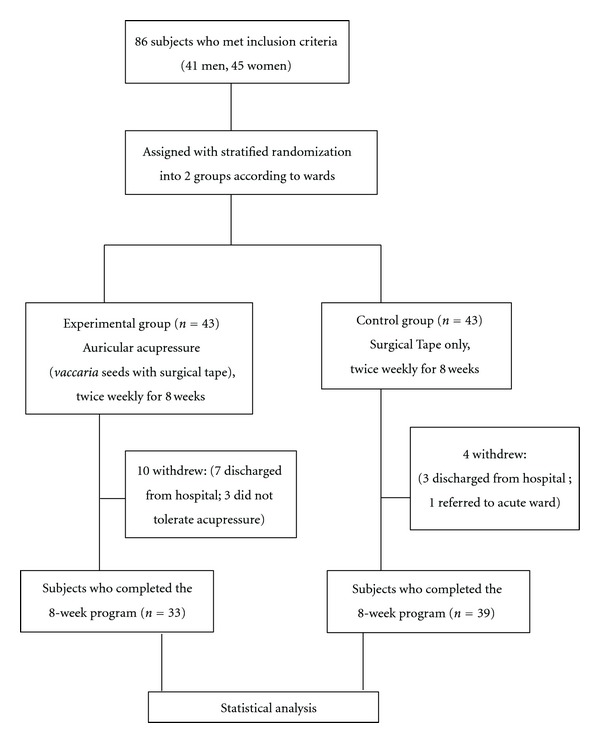
Study flowchart.

There were 86 patients (41 men [48%] and 45 women [52%]) who were recruited and assigned by stratified randomization according to the wards. Each patient was assigned a sequence number according to the medical record number, and then random numbers were obtained from a random number table to divide the patients into 2 groups (43 patients in each group): (1) experimental auricular acupressure group and (2) control group (Figure 1).

During the study period, patients maintained their normal daily lives and were not required to manage diet and exercise themselves to control weight.

Inclusion criteria were meeting the DSM-IV criteria for schizophrenia, staying in a chronic psychiatric ward for >2 months, and meeting the following criteria: (1) age between 20 and 60 years; (2) body mass index (BMI) ≧24 kg/m^2^; (3) current stable mental status and able to communicate with researchers by written or verbal communication.

Exclusion criteria were (1) a person was determined by a court to be incapable of consistently making decisions about his person and his property or some part of either; (2) endocrine disorders: such as abnormal function of thyroid, pituitary, and sex glands; (3) heart diseases: such as arrhythmia, myocardial infarction, heart failure, or installed pacemaker; (4) immune and allergic diseases: such as systemic lupus erythematosus and asthma; (5) liver or kidney dysfunction: GOT or GPT > 80 IU/L, Cr > 2.5 mg/dL; (6) pregnant or lactating women; (7) <6 months postpartum; (8) physical dysfunction because of stroke; (9) involvement in any weight control program within the previous 3 months; (10) determined by the attending psychiatrist to be unsuitable for participation, because of flare ups of psychosis or risk of violence or self-harm.

### 2.1. Experimental Design

The participants were randomly divided into a treatment and a control group, that measurements would take place weekly and that pre- and posttreatment data would be compared.

To improve data objectivity, auricular acupressure was performed and main outcome measures were determined by different persons. There were 6 staff members who were responsible for collecting effect indicators. To increase reliability of data collection, the interrater agreement on waist circumference was assessed from March 8 to 12, 2010; after 6 raters measured 20 patients for waist circumference, the interrater reliability of the results were computed by Pearson correlation analysis. Through communication and training, the interrater reliability reached 0.95 to 1.0 among the 6 raters.

The weight control program was conducted over 9 weeks (including one pretest and 8 subsequent tests, at 1-week intervals); the follow-up time between the 2 groups was conducted as follows for the effect indicators: (1) body weight and waist circumference were measured weekly 9 times in a time series, including once at 1 week before the intervention and weekly through the first to the eighth weeks after the intervention; (2) body fat percentage was measured only 1 week before the intervention and at 8 weeks after the intervention. The study flowchart is shown in Figure 1.

### 2.2. Auricular Acupressure Group

Auricular acupressure was performed by a licensed acupuncturist, who had 540 hours of acupuncture training before participating in the trial. The checklist of consolidated standards of reporting trials (CONSORT) was complete [25]. The complete details of the intervention are presented in Table 1 in conformance to standards for reporting intervention in clinical trials of acupuncture (STRICTA 2010) [26]. Auricular acupressure was given twice weekly for 8 weeks; auricular acupressure was administered to each ear and left in place for 3 and 4 consecutive days alternately. *Vaccaria* seeds with Surgical Tape (3M, Taiwan) were applied at 4 acupuncture points, including the hunger, stomach, shenmen, and endocrine points on the ear. Acupuncture points were selected based on previous studies and clinical experience. Patients were instructed to press on the *Vaccaria segetalis* plaster at each acupuncture point before consuming each of 3 meals every day (4 minutes total, 1 minute for each acupuncture point until the ear had a burning sensation). Compliance with self-pressure at the acupuncture points was monitored with 2 methods: (1) nursing staff of the chronic psychiatric wards reminded and monitored patients while they pressed the acupuncture points at morning, noon, and evening meals and (2) researchers provided a form on which each patient recorded the time of self-application of acupressure.

### 2.3. Control Group

In the control group, Surgical Tape was applied twice weekly, for 3 and 4 consecutive days, for 8 weeks. Selected acupuncture points were the same as those for the AA group, but only Surgical Tape was applied and no points were pressed. The contact moment was just comparable to that in the AA group. The same acupuncturist applied the Surgical Tape in the control group.

### 2.4. Body Composition Measurement

Inpatients received controlled meals from the central kitchen in the hospital. Body weight and waist circumstance were measured 2 hours after dinner. A night snack and drink were served 2.5 hours after dinner. All patients had weight parameters measured before having the night snack and drink.

For the measurement, patients were asked to wear only underwear. The scale precision was calculated as ±0.1 kg. The BMI was calculated by dividing weight in kilograms by the square of height in meters. The measuring tape was placed around the waist at the level of the umbilicus. The tape was held horizontally and close to the skin without disturbing breathing. At the end of expiration, the waist circumference was measured with a precision of ±0.1 cm.

Body fat percentage was measured 2 hours after meals with a body fat analyzer, based on bioelectrical impedance analysis (Type Tanita-519, Japan). Patients were asked to urinate before measurement.

### 2.5. Statistical Analysis

Data were entered in an Excel worksheet and were analyzed with SPSS Statistical Software (version 14.0) (SPSS, Chicago, IL, USA), for descriptive statistics (percentage, mean, standard error, and 95% confidence interval) and analytical statistics (chi-square test, ANOVA, and generalized estimation equation; GEE) [27].

## 3. Results

All 33 experimental patients and 39 control patients completed the auricular acupressure or control treatment for each week. There were 39 women (54.1%) and 33 men (45.9%); 51 (70.8%) patients did not join any sheltered workshops in the hospital. In 72 patients, there were 57 patients (79.2%) taking second generation antipsychotics (SGA), 20 patients (27.8%) taking Clozapine in dosage between 225 and 600 mg daily, 18 patients (25%) taking Risperidone in dosage between 4 and 8 mg daily, 8 patients (11.1%) taking Olanzapine in dosage between 5 mg and 20 mg daily, 6 patients (8.3%) taking Zotepine in dosage between 100 mg and 300 mg daily, 4 patients (5.5%) taking Amisulpride in dosage between 400 mg and 1200 mg, and 1 patient (1.4%) taking 10 mg Aripiprazole daily; there were 15 patients (20.8%) taking first generation antipsychotics (FGA), 8 patients (11.1%) taking Haloperidol in dosage between 10 mg and 20 mg daily, 5 patients (6.9%) taking sulpiride in dosage between 600 mg and 1200 mg daily, 2 patients (2.8%) taking 30 mg Trifluoperazine daily. There were 8 patients (11.1%) taking mood stabilizer with SGA or FGA (Lithium: 2, Valproic acid: 4, Lamotrigine: 2), 3 patients (4.2%) taking SGA with selective serotonin reuptake inhibitor (SSRI), 3 patients (4.2%) taking 2 types of SGA, and 4 subjects (5.5%) taking SGA with FGA. The mean body height was 160.6 ± 1.5 cm in the AA group, 161.2 ± 1.1 cm in the control group; mean age was 46.8 ± 1.6 years in the AA group, 48.6 ± 1.4 years in the control group; mean disease duration of schizophrenia was 15.9 ± 0.9 years in the AA group, 14.2 ± 0.9 years in the control group; mean onset age of schizophrenia was 30.9 ± 1.3 years in the AA group, 34.4 ± 1.8 years in the control group; mean length of hospitalization was 5.6 ± 0.6 years in the AA group, 4.9 ± 0.6 years in the control group.

There was no difference in sex, joining a sheltered workshop (occupational training), or use of second generation antipsychotics between the AA group and control group (Table 2). There was no difference between the AA group and control groups in mean body height, age, disease duration, age at onset of schizophrenia, or length of hospitalization (Table 3).

In waist circumference and body weight, there was no significant difference between the 2 groups at pretest (week 0), and no significant difference from week 1 to 8 compared with week 0 in the control group, and no significant difference between the slopes of AA group and control group from week 1 to week 8 (Tables 4 and 5). No significant differences were shown in body fat percentage between the two groups at pretest; no significant differences were found between pre- and posttest body fat percentage in the control group.

During the study, 10 experimental patients withdrew from the study; 7 patients refused to continue and 3 patients could not perform the auricular acupressure or reliably record a treatment form. 4 control patients withdrew from the study; 3 patients refused to continue and 1 patient was referred to an acute ward because of relapse of psychosis.

3 experimental patients and 1 control patient experienced skin itch after applying plasters on the ear for 2–4 days. The symptom improved and disappeared completely after sticking the plaster on the contralateral ear. And no one withdrew the study because of skin itch.

## 4. Discussion

The present study showed that auricular acupressure did not reduce body weight parameters significantly in patients with chronic schizophrenia. All participants were inpatients, and factors relating to diet, and activity level were better controlled than in outpatient studies. It had been suggested that auricular acupressure may stimulate the sympathetic nervous system, and cause a temporary increase in basal metabolic rate and decrease in appetite that would resolve after the second week [28]. The failure of auricular acupressure to decrease body weight may be a result of the effects of antipsychotic medication. These drugs were required for treatment, and 80% patients treated with antipsychotic medication experience medication-induced weight gain [29]. Second-generation antipsychotics have fewer extrapyramidal adverse reactions than first-generation antipsychotics, but they increase body weight and risk of comorbidities such as hypertension, coronary heart disease, diabetes, and stroke [30, 31].

For example, weight gains for patients treated with Clozapine, Olanzapine, and Risperidone were 4.5, 4.2, and 2.1 kg, respectively, over 10 weeks of treatment [32]. Additional studies showed that the use of Aripiprazole for 1 year caused a mean weight gain of approximately 1 kg [33].

The American Diabetes Association reported that Clozapine and Olanzapine were associated with the greatest potential for weight gain, with evidence of increased risk of diabetes and dyslipidemia. Risperidone was associated with a less potential for weight gain, with discrepant results concerning the risk of diabetes and dyslipidemia. Aripiprazole was associated with minimal weight gain, with no evidence of risk for diabetes and dyslipidemia [34]. A review of Zotepine studies reported a mean body weight gain of 3.6 kg and that 28% of Zotepine-treated patients experienced body weight gain [35]. There were limited published data examining the possible association between Zotepine therapy and the development of diabetes, hyperglycemia, and dyslipidemia. A pooled analysis of data reported an estimated mean weight gain of 0.8 kg with Amisulpride after 10 week of treatment [35], this limited weight gain potential predicts that Amisulpride may be associated with a low risk of adverse metabolic events. The mechanisms may be related to several neurotransmitters, including serotonin, histamine, and dopamine and the adrenergic and muscarinic systems [36, 37].

In addition to medical treatment, all patients received supportive psychotherapy, family therapy, and a series of curriculums on psychiatric rehabilitation, including social skills training, self-care training, and psychoeducation on drug compliance.

Referring to dropouts and withdrawals it has to be mentioned that the Chinese Dragon Boat Festival conflicted with the study schedule, 7 patients in the AA group and 3 patients in the control group were discharged for family gathering. 3 patients in the AA group could not perform the auricular acupressure or record the form by themselves reliably, possibly due to cognitive function decline caused by the mental illness.

There was one patient in the control group who withdrew from the study due to flare up of psychosis (Figure 1), and was transferred from chronic psychiatric ward to acute psychiatric ward which was not related to the use of Surgical Tape. It happens frequently in the chronic psychiatric ward and previous studies have revealed that significant predictors for the relapse of schizophrenia are the number of previous hospitalizations and the number of different antipsychotics previously used [38].

The ear skin itch was related to the use of the Surgical Tape, for it happened in both AA group and control group. According to the classification of WHO's Adverse Reaction Terminology (WHO-ART) [39], the adverse reaction was classified as time-related type. The symptom improved and disappeared after sticking the Surgical Tape on the contralateral ear. We chose the Surgical Tape due to its high viscosity, preventing the tape from sliding off the ear. It is suggested that future studies should consider both the viscosity and antianaphylaxis before performing auricular acupressure.

The traditional Chinese medicine syndrome, a profile of symptoms and signs, is important for understanding human homeostasis and guiding the application of Chinese herbs and acupuncture [40]. Damp stasis syndrome (excess) and Qi deficiency syndrome (deficiency), are common syndromes in obese patients. The excess syndrome includes accumulation or stagnation of metabolic waste, body fluids, and blood, and the deficiency syndrome includes weakness and the deficiency of nutrients [40]. Depending on the symptoms and signs, different patients may be given different traditional Chinese medicine treatments, even when they have the same clinical diagnosis. Future studies may include auricular acupressure according to the differentiation of traditional Chinese medicine syndrome and the needs of each patient.

In the present study, we adopted random assignment by wards primarily to control the 2 confounding factors, diet and exercise, which could greatly affect body weight. The diets in each ward were similar, and it was assumed that similar diet and exercise frequency or intensity were a feature of all the wards. Other confounding factors such as sex, age, and medications were controlled.

Limitations of the present study included the small sample size, which enabled only 2 study arms, and a sham group was not included. Furthermore, the antipsychotic medication were classified only as typical (first generation) and atypical (second generation) drug therapies. The single-blind study design could not be extended to a double-blind design because the AA group patients could easily become aware that the seeds of *Vaccaria segetalis* were contained in the plaster when they performed acupressure. Furthermore, the baseline BMI in the first intervention episode could not be obtained retrospectively.

## 5. Conclusion

Auricular acupressure had no demonstrated efficacy in controlling body weight and waist circumference in patients with chronic schizophrenia. Applying the principles of traditional Chinese medicine, future studies may evaluate auricular acupressure according to the differentiation of traditional Chinese medicine syndrome and each subject's individual needs.

## Figures and Tables

**Table 1:** Standards for reporting interventions in clinical trials of acupuncture (STRICTA 2010).

Acupoint rationale	(1) According to meridian theory of traditional Chinese medicine
	(2) Ear acupoints (hunger, stomach, shenmen, and endocrine)

Acupressure detail	(1) 4 *vaccaria* seeds with Surgical Tape
	(2) Bilateral (each ear acupoints for 3 and 4 consecutive days alternately)
	(3) Pressure feeling
	(4) Burning sensation of the ear
	(5) Manual acupressure
	(6) 4 minutes each time (1 min for each acupuncture point)
	(7) Crude *vaccaria* seeds, diameter of *vaccaria* seed = 2 mm

Treatment regimen	(1) 16 sessions (twice per week)
	(2) Duration, 8 weeks duration

Cointerventions	(1) None of herbs, moxibustion, cupping, massage, exercise, dietary advice, or lifestyle modification

Practitioner background	Licensed Chinese medical doctor, who has had 540 hours of acupuncture training

Control intervention	(1) Same ear acupoints
	(2) Surgical Tape
	(3) No acupressure

**Table 2:** Comparison of control variables and dependent categorical variables between the two groups.

Variable	Group		*χ* ^2^ value	*P* value
	AA group	Control group		
Sex				
Men	15 (46)	18 (46)	0.000	1.000
Women	18 (54)	21 (54)		
Join sheltered workshop				
Yes	10 (30)	11 (28)	0.000	1.000
No	23 (70)	28 (72)		
Secondgeneration antipsychotics				
No	9 (27)	6 (15)	0.896	0.344
Yes	24 (73)	33 (85)		

AA group: experimental group, auricular acupressure with *vaccaria* seeds and Surgical Tape; control group: Surgical Tape only.

**Table 3:** Comparison of control variables and dependent continuous variables between the two groups.

	Number	Mean	Standard deviation	95% confidence interval for the mean		*F* value	*P* value
				L	U		
Body height (cm)							
AA group	33	160.6	1.5	157.6	163.6	0.111	0.740
Control group	39	161.2	1.1	158.9	163.5		
Age (y)							
AA group	33	46.8	1.6	43.6	50.0	0.729	0.396
Control group	39	48.6	1.4	45.7	51.4		
Disease duration (y)							
AA group	33	15.9	0.9	14.0	17.7	1.584	0.212
Control group	39	14.2	0.9	12.4	16.1		
Onset age (y)							
AA group	33	30.9	1.3	28.2	33.6	2.287	0.135
Control group	39	34.4	1.8	30.8	37.9		
Length of hospitalization (y)							
AA group	33	5.6	0.6	4.4	6.8	0.768	0.384
Control group	39	4.9	0.6	3.6	6.1		

AA group: experimental group, auricular acupressure with *vaccaria* seeds and Surgical Tape; control group: Surgical Tape only.

**Table 4:** Changes in mean waist circumference, body weight, and body fat percentage.

Time	AA group (*n* = 33)			Control group (*n* = 39)		
	i1	i2	i3	i1	i2	i3
Week 0						
Mean	95.9	71.4	32.9	95.9	71.7	34.1
sd	7.6	8.6	8.7	6.5	6.8	7.4
Week 1						
Mean	96.9	71.2		98	72.2	
sd	7.8	8.5		7	7.4	
Week 2						
Mean	95.8	71.4		95.7	71.7	
sd	8.3	8.9		6.6	7.3	
Week 3						
Mean	96.7	71.1		96.6	71.6	
sd	7.8	8.8		6.4	7.1	
Week 4						
Mean	96.1	71.3		96.4	72	
sd	8	8.7		6.7	6.9	
Week 5						
Mean	95.5	71.2		96.2	71.7	
sd	8.1	8.9		6.8	7	
Week 6						
Mean	95.1	70.8		95.8	71.6	
sd	8.4	8.7		6.7	6.8	
Week 7						
Mean	96.3	71.2		96.9	71.5	
sd	7.5	8.7		6.4	6.8	
Week 8						
Mean	94.8	70.7	33.2	95.2	71.2	33.1
sd	8.1	8.6	8.9	6.6	6.5	7.1

AA group: experimental group, auricular acupressure with *vaccaria* seeds and Surgical Tape; Control group: Surgical Tape only; i1: waist circumference; i2: body weight; i3: body fat percentage.

**Table 5:** Comparison of intervention effects between the two groups.

Variables	Regression coefficient	standard error	*t* value	*P* value
Waist circumference (cm)				
Control group at week 0	95.91			
Week 0 (AA group/control group)	−0.04	1.72	−0.03	0.979
Control group (week 1/week 0)	2.09	1.67	1.25	0.211
Control group (week 2/week 0)	−0.20	1.64	−0.12	0.905
Control group (week 3/week 0)	0.69	1.64	0.42	0.676
Control group (week 4/week 0)	0.52	1.64	0.31	0.754
Control group (week 5/week 0)	0.29	1.64	0.18	0.859
Control group (week 6/week 0)	−0.09	1.64	−0.06	0.954
Control group (week 7/week 0)	0.98	1.64	0.60	0.552
Control group (week 8/week 0)	−0.72	1.64	−0.43	0.664
Difference of slopes from week 0 to week 1 between 2 groups	−1.05	2.47	−0.42	0.671
Difference of slopes from week 0 to week 2 between 2 groups	0.10	2.43	0.04	0.968
Difference of slopes from week 0 to week 3 between 2 groups	0.18	2.43	0.07	0.942
Difference of slopes from week 0 to week 4 between 2 groups	−0.30	2.43	−0.12	0.902
Difference of slopes from week 0 to week 5 between 2 groups	−0.63	2.43	−0.26	0.796
Difference of slopes from week 0 to week 6 between 2 groups	−0.70	2.43	−0.29	0.773
Difference of slopes from week 0 to week 7 between 2 groups	−0.49	2.43	−0.20	0.840
Difference of slopes from week 0 to week 8 between 2 groups	−0.36	2.43	−0.15	0.882
Body weight				
Control group at week 0	71.68	1.25	57.31	
Week 0 (AA group/control group)	−0.27	1.85	−0.14	0.885
Control group (week 1/week 0)	0.54	1.79	0.30	0.761
Control group (week 2/week 0)	0.06	1.77	0.03	0.972
Control group (week 3/week 0)	−0.06	1.77	−0.03	0.972
Control group (week 4/week 0)	0.28	1.77	0.16	0.876
Control group (week 5/week 0)	0.06	1.77	0.03	0.972
Control group (week 6/week 0)	−0.12	1.77	−0.07	0.945
Control group (week 7/week 0)	−0.20	1.77	−0.11	0.910
Control group (week 8/week 0)	−0.47	1.77	−0.27	0.790
Difference of slopes from week 0 to week 1 between 2 groups	−0.80	2.65	−0.30	0.763
Difference of slopes from week 0 to week 2 between 2 groups	−0.10	2.61	−0.04	0.969
Difference of slopes from week 0 to week 3 between 2 groups	−0.28	2.61	−0.11	0.915
Difference of slopes from week 0 to week 4 between 2 groups	−0.38	2.61	−0.14	0.885
Difference of slopes from week 0 to week 5 between 2 groups	−0.28	2.61	−0.11	0.915
Difference of slopes from week 0 to week 6 between 2 groups	−0.46	2.61	−0.18	0.861
Difference of slopes from week 0 to week 7 between 2 groups	−0.05	2.61	−0.02	0.983
Difference of slopes from week 0 to week 8 between 2 groups	−0.23	2.61	−0.09	0.929
Body fat percentage				
Control group at week 0	34.15	1.28	26.66	
Week 0 (AA group/control group)	−1.24	1.89	−0.66	0.512
Control group (week 8/week 0)	−1.01	1.81	−0.56	0.577
Difference of slopes from week 0 to week 8 between 2 groups	1.26	2.68	0.47	0.638

AA group: experimental group, auricular acupressure with *vaccaria* seeds and Surgical Tape; control group: Surgical Tape only.
